# Sandwich-Structured h-BN/PVDF/h-BN Film With High Dielectric Strength and Energy Storage Density

**DOI:** 10.3389/fchem.2022.910305

**Published:** 2022-07-04

**Authors:** Guodong Meng, Junyi She, Changling Wang, Wenke Wang, Cheng Pan, Yonghong Cheng

**Affiliations:** ^1^ State Key Laboratory of Electrical Insulation and Power Equipment, Xi’an Jiaotong University, Xi’an, China; ^2^ School of Electrical Engineering, Wuhan University, Wuhan, China

**Keywords:** sandwiched-structure, Ultrathin hexagonal boron nitride, polyvinylidene fluoride, energy storage density, dielectric Strength

## Abstract

Energy storage film is one of the most important energy storage materials, while the performance of commercial energy storage films currently cannot meet the growing industrial requirements. Hence, this work presents a h-BN/PVDF/h-BN sandwich composite structure film prepared by laminating a large area of ultrathin hexagonal boron nitride (h-BN) and polyvinylidene fluoride (PVDF), the existence of which was confirmed by using an optical microscope and elemental composition analysis based on scanning electron microscopy and X-ray diffraction. This film has an ultrahigh dielectric strength of 464.7 kV/mm and a discharged energy density of up to 19.256 J/cm^3^, which is much larger than the commercial energy storage film biaxially oriented polypropylene (BOPP) (<2.5 J/cm^3^). Although the thickness of the h-BN film is only 70 nm compared with that of PVDF (about 12 *μ*m), the dielectric strength of the sandwich-structured film presents a great increase. It is because of the excellent insulation performance of the h-BN film that helps to resist the electron injection and migration under high electric field, and then suppress the formation and growth of the breakdown path, leading to an improvement of the charge–discharge efficiency.

## Introduction

Nowadays, dielectric capacitors are widely used in the electric power system, integrated circuits, electric vehicles, and other fields due to the high power density, fast charge–discharge rate, large operating voltage, and low dielectric loss. In general, dielectric energy storage materials mainly include ceramic materials and polymer materials. Polymer materials such as polyvinylidene fluoride (PVDF), polypropylene (PP), and poly (ethylene imine) (PEI) have received extensive attention attributed to the high breakdown field strength, light weight, facile processability, and scalability. In particular, as a typical polymer material, PVDF has relatively high permittivity, especially the PVDF in the γ-phase has the smallest hysteresis loss and could work under higher electric fields than other phases, which is often applied in energy storage films. However, the films based on PVDF and other polymer materials could only achieve high energy storage performance under high electric field. In order to meet the requirements of high permittivity, high breakdown field strength, and low dielectric loss under relatively low electric field, the films need to be further modified.

For linear dielectrics, the storage energy density scales linearly with the permittivity of the dielectric materials and scales quadratically with the breakdown electric field strength. As suggested by the following equation:

$$U_{e}=\frac{1}{2}\varepsilon_{0}\varepsilon_{r}E^{2}$$ (1)

where *U*_*e*_ is the storage energy density of the dielectrics, *ε*_*r*_ is the relative permittivity of dielectric material, and *ε*_*0*_ is the permittivity of vacuum. It is obvious that strategies for acquiring higher permittivity and higher breakdown field strength represent an efficient target for the construction of high-performance polymer-based film capacitors.

Based on the theoretical formula (Eq. 1), there are some approaches commonly used to improve the performance of energy storage films including element doping (Ke et al., 2013; Peng et al., 2015; Li et al., 2016), adding high permittivity fillers (Thakur et al., 2017; Ai et al., 2020; Zhang et al., 2020), and laminated structure (Wang and Zhu, 2011; Sun et al., 2018; Sheng et al., 2020). The laminated structure could integrate the complementary properties of spatially organized multiple-components in a synergistic way to improve charge–discharge efficiency and maintain low dielectric loss. For example, Azizi et al. (2017) transferred h-BN onto both sides of the PEI film, and the h-BN–coated films operated with more than 90% charge–discharge efficiency. Chi et al. (2017) selected PVDF doped by CFO@0.5Ba(Zr_0.2_Ti_0.8_)O_3_-0.5(Ba_0.7_Ca_0.3_)TiO_3_ nanofibers as an intermediate layer, and pristine PVDF films as the outer layers, and the maximum discharged energy density is about 11.3 J/cm^3^ and efficiency is about 55.5% at the electric field of 350 kV/mm ). Cheng et al. (2020) fabricated a laminated structure of polyimide (PI) film coated by boron nitride (BN) films, and the sandwiched film could discharge an energy density of 0.493 J/cm^3^, with charge–discharge efficiency of over 90% under an electric field of 200 MV/m.

In this work, two-dimensional hexagonal boron nitride (h-BN) was introduced and a h-BN/PVDF/h-BN sandwich-structured film was prepared. The h-BN film was fabricated by chemical vapor deposition (CVD), and the sandwich-structured film was fabricated by covering both sides of PVDF films using the hot-pressing method. The thickness and microstructure of the sandwich-structured film were characterized by using an optical microscope, scanning electron microscope (SEM), atomic force microscope (AFM), and X-ray diffraction (XRD). The dielectric and energy storage performances of the sandwich-structured film were examined and analyzed, and the influence mechanism of the h-BN coating layers to the PVDF film was discussed.

## Experiment

### Fabrication of PVDF by Solution Volatilization

**FIGURE 1:**
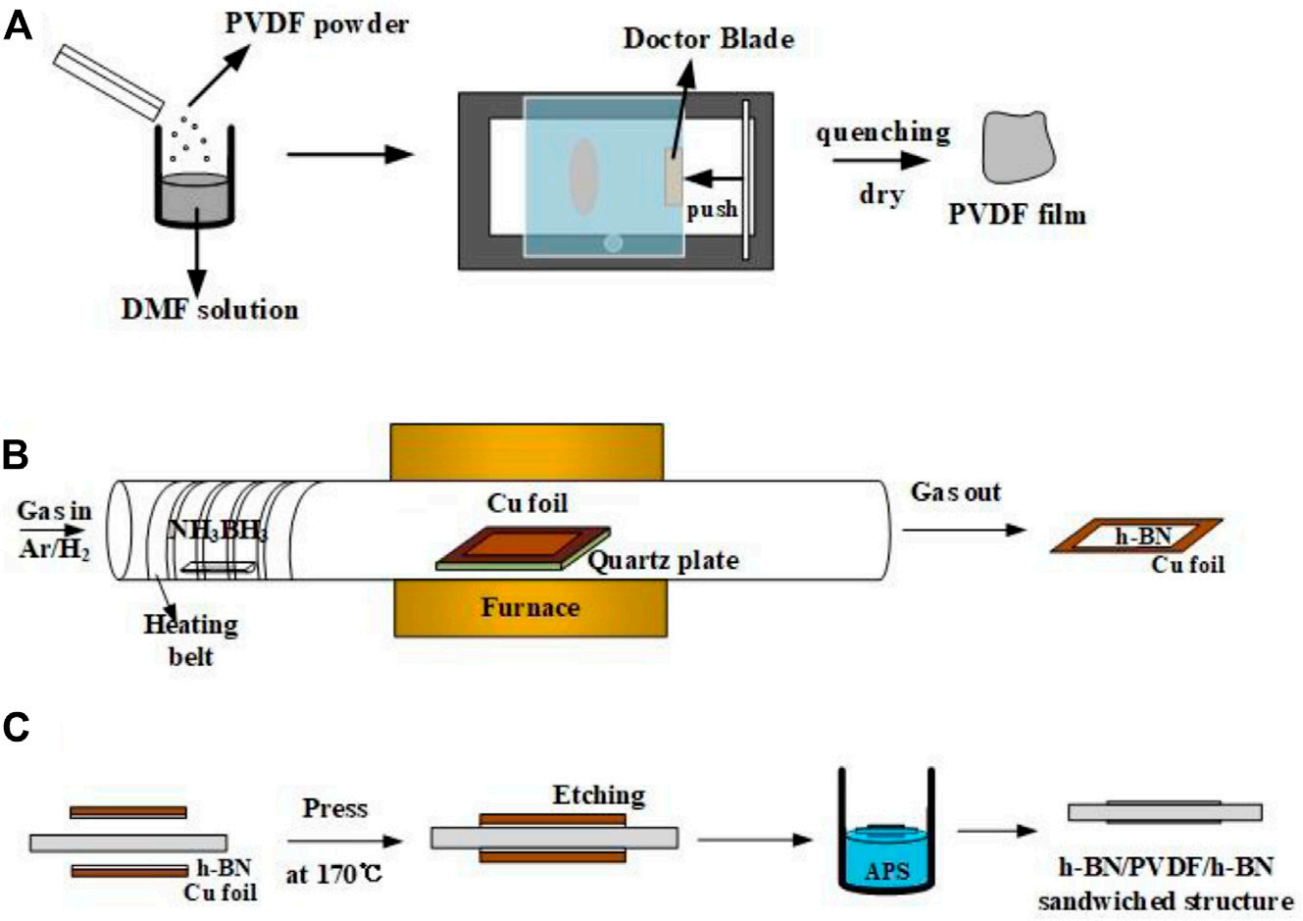
Preparation process of pristine PVDF films **(A)**, h-BN/Cu films **(B)** and h-BN/PVDF/h-BN sandwich-structured films **(C)**.

As shown in Figure 1A, polyvinylidene fluoride (PVDF, the molecular weight is 534,000) powder is dissolved in N, N-dimethylformamide (DMF, the molecular weight is 73.09) solution in a ratio of 1.2–10. The mixed solution is then put into the magnetic stirrer for 2 h at 60°C. The solution is poured evenly on the film coater (MSK-AFA-HC100), and the doctor blade is set to 55 cells (equivalently 12 μm). After fully removing DMF from a drying oven at 60°C, PVDF is heated to 200 °C for 10 min and cooled down rapidly in ice water in order to transform to the γ-phase more thoroughly.

### Fabrication of h-BN by CVD

Common methods for preparing h-BN films include chemical vapor deposition (CVD), mechanical lift-off, ion beam sputtering, and magnetron sputtering deposition. Chemical vapor deposition is better at controlling the thickness and size of films, especially suitable for scale production. As shown in Figure 1B, the h-BN films were synthesized on a Cu foil by the CVD method. The substrates are Cu foils with 25 μm thick, 99.999% pure (Alfa Aesar, part #10950), which were cleaned by isopropanol and then loaded into a furnace; 2.2 g ammonia borane (NH_3_BH_3_, Aldrich) was used as the precursor and placed in the front of the quartz tube being heated by a heating belt. In order to maintain a low growth pressure, a vacuum pump was connected to the end of the quartz tube to maintain the pressure below 1 Torr during the whole process. Before the growth, 20 sccm hydrogen and 200 sccm argon were passed into the tube. Cu foils were put into the surface and heated to a temperature of 1,000 °C and maintained for 1 h in hydrogen/argon atmosphere. The purpose of this operation is to remove the organics and oxide attached to the surface of Cu foils. During the growth, temperature is maintained at 1,000°C, and ammonia borane was heated to 120 °C for about 30 min.

### Fabrication of h-BN/PVDF/h-BN Sandwiched-Structure by Hot Pressing

In this work, two pieces of h-BN/Cu films and one piece of PVDF film are stacked neatly to form a sandwiched-structure (Figure 1C). The structure was fabricated by hot pressing under 5 MPa at 170°C, temperature that is close to the glass transition temperature (T_g_) of PVDF, for 20 min. Then, the Cu substrates from both sides were etched by ammonium persulfate solution (APS, 0.3 mol/L). The laminate structure was left floating in the etchant solution for 30 min at 40°C, until all of Cu was dissolved into the solution. Then, the sandwiched-structure was rinsed with deionized water for 10 min to remove the residual etchant. Finally, the sample was removed from deionized water and left in air for 1 h to dry.

## Results and Discussion

### Structural Characterization

**FIGURE 2:**
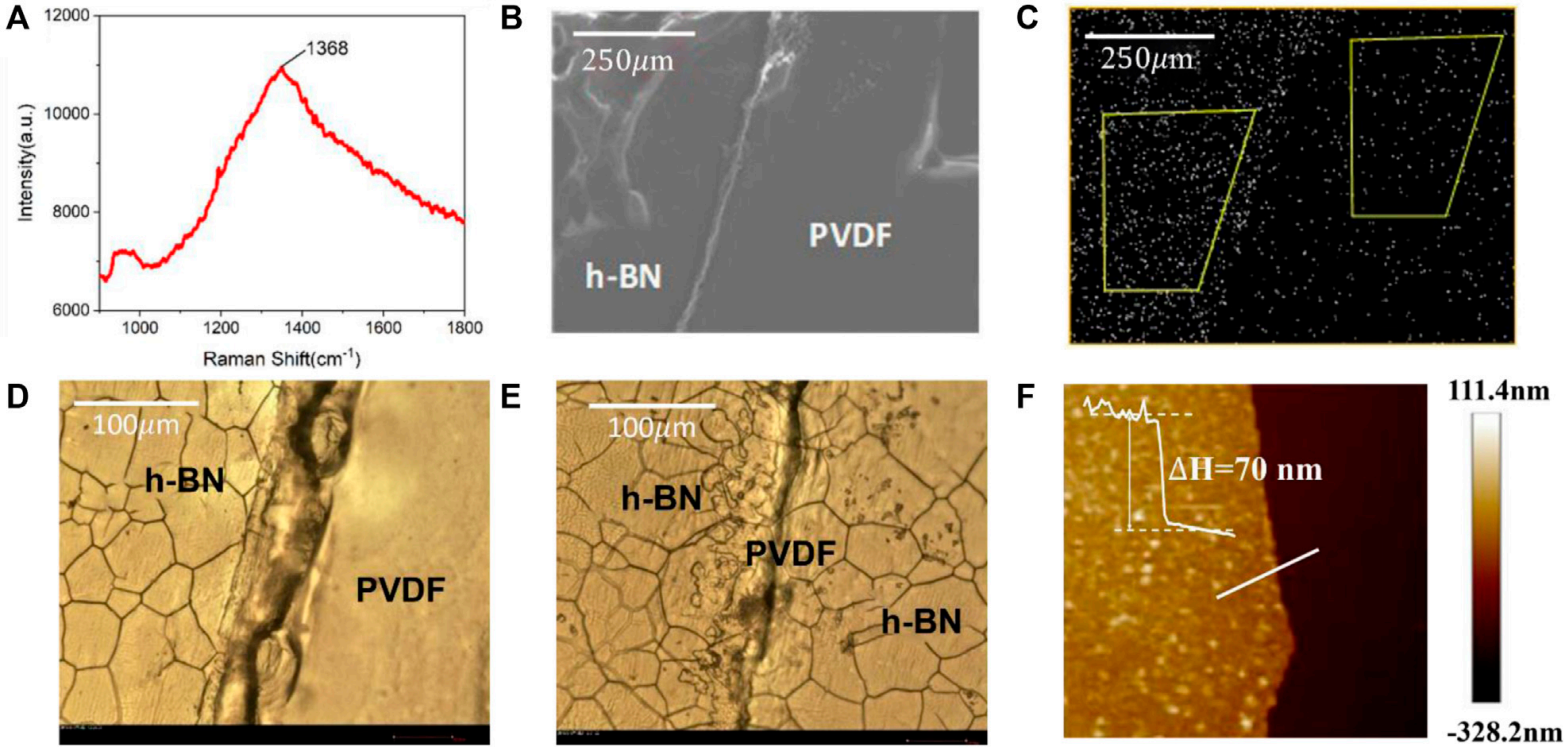
Raman spectrum of the h-BN film on Si/SiO_2_/substrate **(A)**. SEM image of h-BN/PVDF/h-BN sandwiched-structure **(B)**. Energy-dispersive spectrometer (EDS) of nitrogen distribution **(C)**. Optical micrograph of single-layer h-BN **(D)**. Optical micrograph of h-BN/PVDF/h-BN structure **(E)**. AFM image of the h-BN/Si film **(F)**.

The characterization results of a h-BN/PVDF/h-BN film on the Raman and SEM-EDS systems are illustrated in Figures 2A–C. Figure 2A presents the Raman spectrum of h-BN films on Si/SiO_2_ substrate, which is recorded using a Horiba LabRAM HR Evolution Raman spectrometer. The Raman spectrum of h-BN films demonstrates a characteristic peak at 1,368.7 cm^−1^ corresponding to the E_2g_ vibration mode of the hexagonal B–N bonds. Figure 2B illustrates SEM image of the interface, which can be distinctly identified between the two materials. As shown in Figure 2C, the nitrogen element presents large differences in distribution on both sides of the dividing line, which indicates that a laminated structure was fabricated.

The samples are further characterized using an optical microscope (BX51-P) and AFM (Bruker Dimension Icon). Since h-BN covers the surface of the Cu foil during the growth process, in order to facilitate AFM testing, the h-BN film is transferred to a Si substrate. Figure 2D presents the boundary of a single-layer h-BN film. The evidently cracked h-BN film can be seen on the left, the flat surface of the PVDF film can be recognized on the right, and the traces in the middle are folds formed by hot pressing. Figure 2E shows the boundary of h-BN/PVDF/h-BN sandwiched-structure. Compared with the single-layer h-BN film, a clear double-layer crack can be found, proving the h-BN/PVDF/h-BN structure. The AFM image of the h-BN/Si film is shown in Figure 2F, the light-colored area is the h-BN film, and the dark area is the silicon substrate. The thickness information on the white line can be obtained from right. It can be seen that the h-BN film is relatively flat, and it can be considered that the average thickness of the prepared h-BN is 70 nm.

### Dielectric Performance Characterization

**FIGURE 3:**
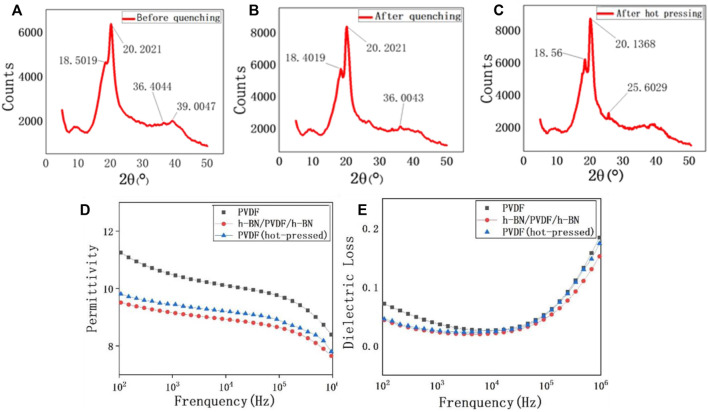
XRD patterns of the PVDF film before quenching **(A)**, after quenching **(B)**, and after hot pressing **(C)**. Frequency-dependent permittivity of pristine PVDF, h-BN/PVDF/h-BN, and hot-pressed PVDF films **(D)**. Frequency-dependent dielectric loss of pristine PVDF, h-BN/PVDF/h-BN, and hot-pressed PVDF films **(E)**.

During the fabrication process of PVDF, the crystal phase transition process is shown in Figures 3A–C. It has been proven that the diffraction peaks at 18.55° and 20.20° reflected from (020) planes and (110)/(101) crystallographic planes can be used for the determination of the γ-phase. Furthermore, the diffraction peaks at 35.95° and 39.10° are identified as (100) of α form overlapped with (200) of α form and the (004) of the γ-phase, respectively (Jurczuk et al., 2015). In Figure 3A, the peaks at 39.0047° confirm the existence of γ-phase, but there is still a considerable number of α-phase, reflected by the peaks at 36.4° and 39.0°. When the 200 °C PVDF is quenched in ice water, more α-phase begins to transform into γ-phase, which can be proven by the relative height of the peak at 18.5° (Figure 3B). However, the presence of 36.0° peak indicates that there is still a small quantity of α-phase after quenching. The hot-pressing process can be regarded as a secondary treatment for the transformation of the PVDF crystal form. After the second treatment, the crystal form of PVDF has completed a thorough transformation (Figure 3C), three peaks at 18.5°, 20.1°, and 25.6° all illustrate that the purity of the γ-phase is significantly higher than the previous one.

The dielectric properties of pristine PVDF, hot-pressed PVDF, and h-BN/PVDF/h-BN films are presented in Figures 3D, E. As expected, both permittivity and dielectric loss have declined. For example, ε_r_ is experimentally found to be 10.5 of PVDF and 9.7 of h-BN/PVDF/h-BN at 1 kHz. An explanation for the reduction in permittivity of the sandwiched structure is that the permittivity of pure α-phase is higher than that of the pure γ-phase. The dielectric loss of the two samples is shown in Figure 3E. At 1 kHz, the dielectric loss factor reduced from 0.036 to 0.024, showing a drop of 30%. The reduction of dielectric loss will contribute to a decrease of energy loss during the process of charging and discharging, improving the efficiency slightly, and as is quantified experimentally, the discharged energy density reached 19.256 J/cm^3^ with charge–discharge efficiency of 52.2%.

### Energy Storage Performance Characterization

**FIGURE 4:**
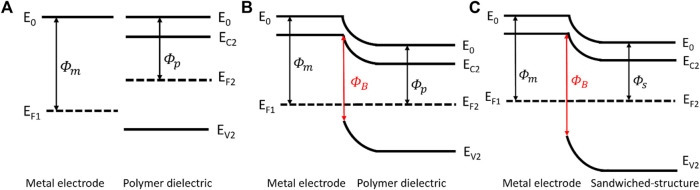
Energy band diagram of metal electrode and polymer dielectric before contact **(A)**. Energy band diagram at metal electrode/polymer dielectric interface **(B)**. Energy band diagram at metal electrode/sandwiched structure interface **(C)**. $\Phi_{m}$ , $\Phi_{p}$ , and $\Phi_{s}$ is the work function of metal electrode, PVDF, and h-BN/PVDF/h-BN sandwiched-structure, respectively. $\Phi_{B}$ is the potential barrier induced by band alignment.

The PVDF film is a typical ferroelectric material whose polarization intensity and electric field intensity indicate a nonlinear relationship. Hence, the Polarization Loop & Dielectric Breakdown Test System is used to investigate the characteristics of our samples. Table 1 illustrates the parameters of three kinds of films, the thickness of the films was determined by micrometer, and it is obvious that the breakdown strength of the composite structure is extremely enhanced in comparison with the pristine PVDF film. The sandwiched structure consists of a PVDF film with high polarization intensity serving as the center layer, and h-BN films with high breakdown strength serving as the outer layers. As illustrated in Figures 4A, B, when the metal electrodes come in contact with polymer dielectric, the energy band bending produces a potential barrier in order to make Fermi levels of the two at the same position. Furthermore, the introduction of h-BN layers cause band alignment at the interface of metal electrode/h-BN/PVDF due to the differences in bandgap and electron affinity, leading to a higher potential barrier, as shown in Figure 4C. The higher potential barrier prevents the charge injection from metal electrodes, hinders the formation of conductive channels and improves the breakdown strength, leading to an increase of discharged energy density. Meanwhile, the residual polarization of the composite film declines slightly, which contributes to the rise of discharged energy density as well.

**TABLE 1:** Parameters of three kinds of samples.

Film	Thickness	Electrode	Electrode area	Quantity	Average *E*_b_
h-BN/PVDF/h-BN	(12.4 ± 0.1) µm	Au	0.0352 $cm^{2}$	3	464.7 kV/mm
PVDF (hot-pressed)	(12.2 ± 0.1) µm	Au	0.0352 $cm^{2}$	3	219.7 kV/mm
PVDF	(12.1 ± 0.1) µm	Au	0.0352 $cm^{2}$	3	346.3 kV/mm

**FIGURE 5:**
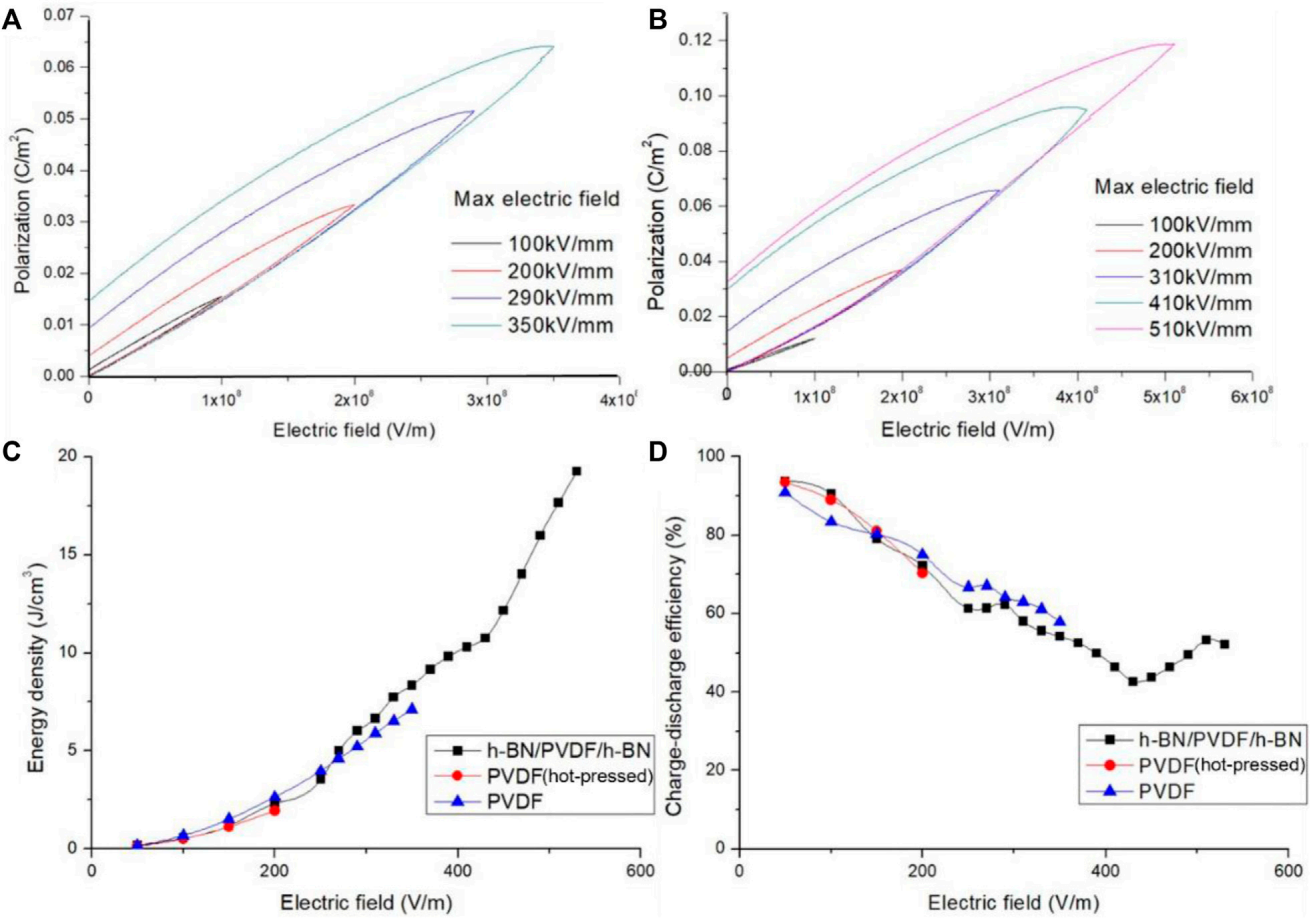
Polarization electric field (P–E) hysteresis of PVDF **(A)** and h-BN/PVDF/h-BN sandwiched-structure **(B)**. Discharged energy density of h-BN/PVDF/h-BN, hot-pressed PVDF, and pristine PVDF films **(C)**. Charge–discharge efficiency of h-BN/PVDF/h-BN, hot-pressed PVDF, and pristine PVDF films **(D)**.

By raising the electric field strength, the maximum polarization of PVDF and sandwiched-structure grows remarkably as illustrated in Figures 5A, B. Induced by an enhancement of the maximum polarization, a better performance of energy storage is obtained in sandwiched-structure. For instance, the maximum polarization of PVDF increases from 0.015203 C/m^2^ at 100 kV/mm to 0.031905 C/m^2^ at 200 kV/mm. Due to the dispersion of data when testing at 100 kV/mm, slopes of polarization loop are different from others. It is found experimentally that the maximum polarization of h-BN/PVDF/h-BN rises to 0.036455 C/m^2^ at 200 kV/mm, more than the value of PVDF under the same conditions, which denotes that it is the use of sandwiched structure that distinctly promote the maximum polarization.

Furthermore, we quantify the discharged energy density (Figure 5C). In the range of 0–200 kV/mm, the discharged energy densities of the three films are low and perform unnoticeable variations. In the range of 200–400 kV/mm, the PVDF film after hot pressing has broken down, and the discharged energy density of the composite film gradually tends to be higher than that of the pristine PVDF film, attributed to the increase of the maximum polarization and the degradation of the residual polarization. In the range of 400–600 kV/mm, the pristine PVDF also broke down, and the discharged energy density of the composite structure reaches 19.256 J/cm^3^ at 510 kV/mm.

As the electric field strength rises, the charge–discharge efficiency (Figure 5D) of the three films gradually declines, and the composite film reduces from 93.8% to 42.7%, then rises back to about 52.2%. In the range of 0–200 kV/mm, the charge–discharge efficiency of the composite film is larger than that of the pristine PVDF films. High potential barriers are achieved in sandwiched structure, which prevents the charges from being injected into the composite film from metal electrodes. As a result, the leakage current and dielectric losses also drop, leading to an improvement of the charge–discharge efficiency. In the range of 200–400 kV/mm, the PVDF after hot pressing has broken down, and the charge–discharge efficiency of the composite film is slightly lower than that of the pristine PVDF. The leakage current and dielectric loss considerably increased due to hot pressing. In the range of 400–600 kV/mm, the pristine PVDF also broke down, and the charge–discharge efficiency of the composite structure is about 50%.

In comparison with similar types of work, some traditional energy storage films based on polymer dielectrics such as PEI, PI, and polycarbonate (PC) have been modified into sandwiched structure. For example, Liu et al. (2020) prepared a h-BN/PC/h-BN sandwich-structured film whose discharged energy density reached 5.52 J/cm^3^. In this work, we selected PVDF with high intrinsic permittivity, which is widely used in filters and AC/DC converters/inverters as the inner layer to fabricate a laminated structure, further enhancing the energy storage performance, and the PVDF film coated by the two-dimensional nanometer h-BN film improved the discharged energy density while maintaining a relatively thin thickness in comparison with other sandwich-structured films with outer layers in bulk materials such as SiO_2_ reported before, which has great potential in integrated circuits. For example, Zhou et al. (2018) deposited 178-nm-thick SiO_2_ onto both sides of biaxially oriented polypropylene (BOPP), and the discharged energy density reached 1.33 J/cm^3^. Thanks to the composite structure of two-dimensional material and polymer dielectric, the sandwiched-structure paves the way for the films with high discharged energy density. At the same time, the combination of sandwiched structure and element doping or high-permittivity fillers will give more possibilities for high-performance energy storage films in the future.

## Conclusion

In order to overcome the large dielectric loss and enhance the breakdown field strength, h-BN films were introduced as insulating layers. The film with higher flatness was prepared by three methods, and the structure of the composite film is satisfying. The breakdown strength and energy storage capability of sandwiched-structure are distinctly enhanced. Compared with a pristine PVDF film, the breakdown field strength of the composite film has increased from an average of 346.3 kV/mm to 464.7 kV/mm. At the same time, the discharged energy density and charge–discharge efficiency under the same conditions still remained relatively high. When applying an electric field of 510 kV/mm, the discharged energy density reached 19.256 J/cm^3^ with the charge–discharge efficiency of 52.2%, indicating that the coating of two-dimensional h-BN films can significantly improve the energy storage properties of the PVDF film.

## Data Availability

The original contributions presented in the study are included in the article/Supplementary Material; further inquiries can be directed to the corresponding author.
